# Patellar malalignment: a new method on knee MRI

**DOI:** 10.1186/s40064-016-3195-0

**Published:** 2016-09-07

**Authors:** Hülya Kurtul Yildiz, Elif Evrim Ekin

**Affiliations:** Radiology Department, Gaziosmanpaşa Taksim Training and Research Hospital, Istanbul, Turkey

**Keywords:** Knee MRI, Medial patellofemoral ligament, Trochlear dysplasia, Patella alta- MPFLL/LPR

## Abstract

**Purpose:**

The medial patellofemoral ligament (MPFLL)/lateral patellar retinaculum (LPR) ratio were assessed in knees as a means to detect patellar malalignment. We also aimed to evaluate the prevalence of the various types of trochlear dysplasia in patients with patellar malalignment.

**Materials and methods:**

After approval of our institutional ethics committee, we conducted a retrospective study that included 450 consecutive patients to evaluate them for the presence of patellar malalignment. Parameters investigated were the trochlear type, sulcus angle, presence of a supratrochlear spur, MPFLL, LPR, patella alta, and patella baja by means of 1.5T magnetic resonance imaging (MRI). Overall, 133 patients were excluded because of the presence of major trauma, multiple ligament injuries, bipartite patella, and/or previous knee surgery. The Dejour classification was used to assess trochlear dysplasia. Two experienced radiologists (HKY, EEE) evaluated the images. Their concordance was assessed using the kappa (κ) test.

**Results:**

The frequencies of patellar malalignment and trochlear dysplasia were 34.7 and 63.7 %, respectively. The frequency of trochlear dysplasia associated with patellar malalignment was 97.2 %. An MPFLL/LPR ratio of 1.033–1.041 had high sensitivity and specificity for malalignment. The researchers’ concordance was good (κ = 0.89, SE = 0.034, *P* < 0.001).

**Conclusion:**

Trochlear dysplasia is frequently associated with patellar malalignment. An increased MPFLL/LPR ratio is useful for detecting patellar malalignment on knee MRI, which is a novel quantitative method based on ligament length.

## Background

Patellar malalignment is defined as an abnormal position of the patella with respect to the femoral trochlear groove in any position (Grelsamer 2005). Patellar malalignment, with lateral tracking of the patella, is held responsible for the patellofemoral pain syndrome, which is a common problem (Doucette and Goble 1992). Important predisposing factors for patellar malalignment are trochlear dysplasia, medial patellofemoral ligamentous laxity, lateral retinacular shortness, patella alta, a tibial tubercle–trochlear groove (TT-TG) distance of >20 mm, and patellar tilt (Bollier and Fulkerson 2011; Arendt and Dejour 2013; Oliveira et al. 2014).

The first line of treatment of patellofemoral malalignment is conservative. When it is decided that surgery is necessary, various combinations of medial patellofemoral ligament (MPFL) reconstruction, lateral release, medial capsular plication, and trochleoplasty can be used (LaPrade et al. 2014). Therefore, preoperative anatomic evaluation is important for the surgical decision and selection of techniques to be used.

To date, the literature has described only evaluations of bony structures. In recent years, the TT-TG distance has been used as the gold standard. To establish this value on magnetic resonance imaging (MRI), however, an additional software program and experience are needed (Hinckel and Gobbi 2015). In this study, we aimed to use a new method for diagnosing patellofemoral malalignment that can be performed using routine MRI evaluation, thereby avoiding the need for the additional cost and experience. Based on the philosophy of the treatment methods, we thought that the length of the ligament could be meaningful for diagnosing patellar malalignment. Therefore, our aim was to apply the medial patellofemoral ligament length/lateral patellar retinaculum (MPFLL/LPR) ratio, which we think is a quick, easy, reliable measurement that could be calculated from routine knee MRI scans. We also evaluated the prevalence of trochlear dysplasia, patella alta, and patella baja in regard to patellar malalignment.

## Methods

### Patient selection

Approval of the local ethics committee was obtained before starting the study. The study population was composed of knee pain and trauma patients referred to our hospital. This retrospective study included 450 consecutive patients who were examined between November 2014 and February 2015. Among them, 133 patients were excluded because of the presence of major trauma, anterior cruciate ligament rupture, multiple ligament injuries, femoral fracture, bipartite patella, previous knee surgery, and/or widespread artifacts. The final analysis included 317 patients.

### MRI techniques

A 1.5-T MRI unit (Signa HDxt; GE Medical Systems, Carrollton, TX, USA) and an extremity coil were used. Sagittal T1-weighted fast spin echo (TR/TE 750/10, matrix size 256 × 256, field of view 18 cm, slice thickness 4 mm, number of excitations 2) and axial proton density (PD) fat-suppressed (TR/TE 4000/40, matrix size 288 × 256, field of view 18 cm, slice thickness 3 mm, number of excitations 2) sequences were used for measurements.

### Evaluation of the images

The frequency of patellar malalignment, trochlear dysplasia, supratrochlear spurs, and patellar height were investigated in patients with patellar malalignment and those with a normal patellofemoral joint. We also studied the types of trochlear dysplasia based on the Dejour classification.

### Patellar malalignment

Detecting patellar malalignment was performed using the qualitative method of Shellock et al. (1989), which is based on the relation between the mediolateral edges of the patella and the femoral trochlear mediolateral sides. In addition, patellar tilt was defined as the angulation between the posterior femoral condylar line and the largest diameter of the patella.

### Sulcus angle and trochlear typing

**Fig. 1:**
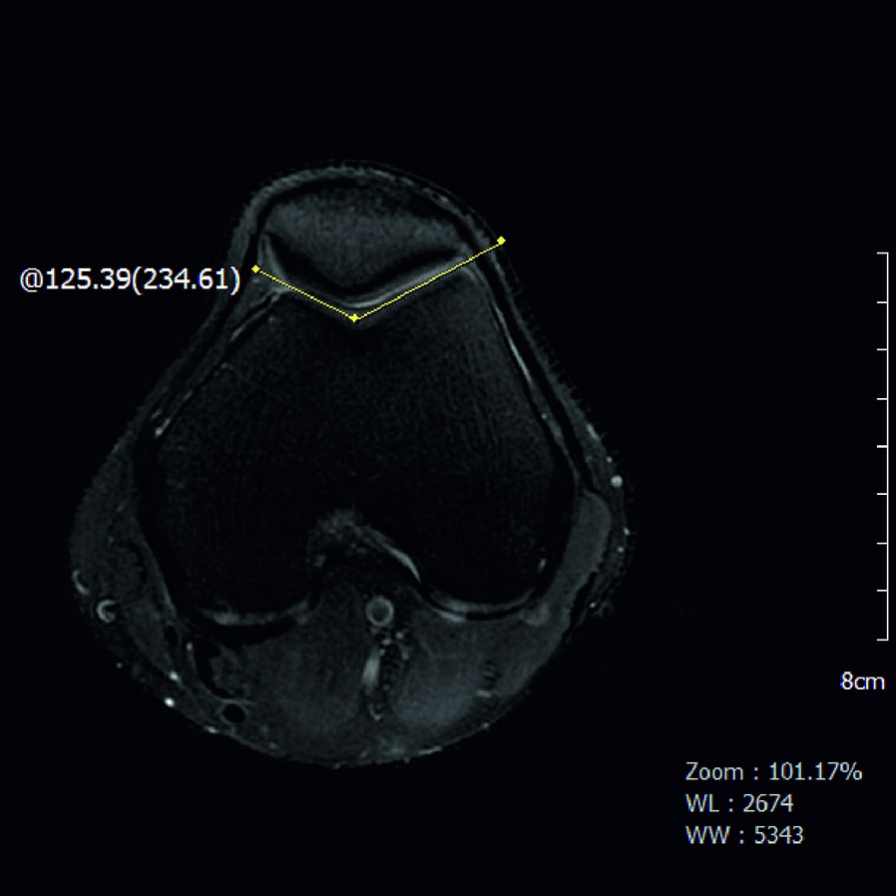
Axial proton density fat-saturated magnetic resonance imaging (PD-fatsat MRI) (a Sect. 3 cm above the knee joint). Note the normal trochlear groove and sulcus angle

Axial plane images >3 cm from the knee joint were used. The sulcus angle was measured from the highest lateral corner on the anterior surface to the deepest sulcus point and then to the highest medial corner. A trochlear angle of 137° ± 8° was accepted as normal (Fig. 1).

The Dejour classification was used to classify trochlear dysplasia. Dejour et al. (1990, 1994) classified trochlear dysplasia based on the trochlear angle and configuration. Dejour suggested the following morphological classification for trochlear dysplasia (Dejour et al. 1990).

**Fig. 2:**
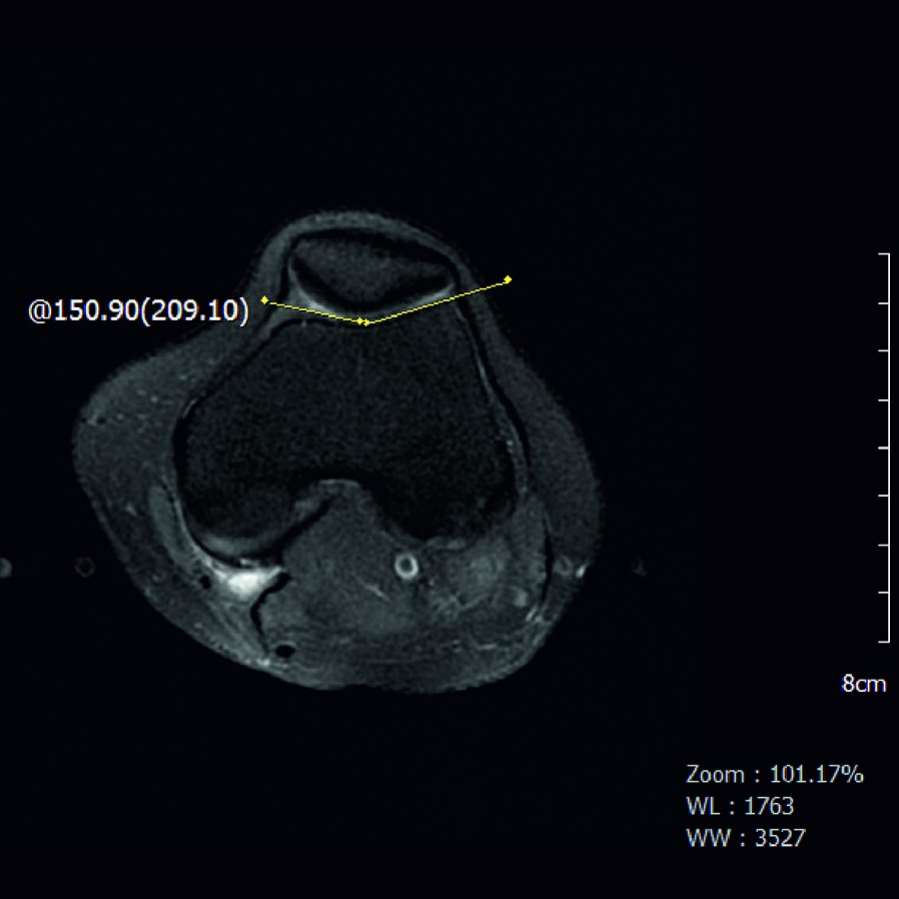
Axial PD-fatsat MRI of a Sect. 3 cm above the knee joint. Although there is type A trochlear dysplasia and the sulcus angle is increased to 150°, the trochlea is symmetric

Type A: sulcus angle >145° but with normal shape (Fig. 2)

**Fig. 3:**
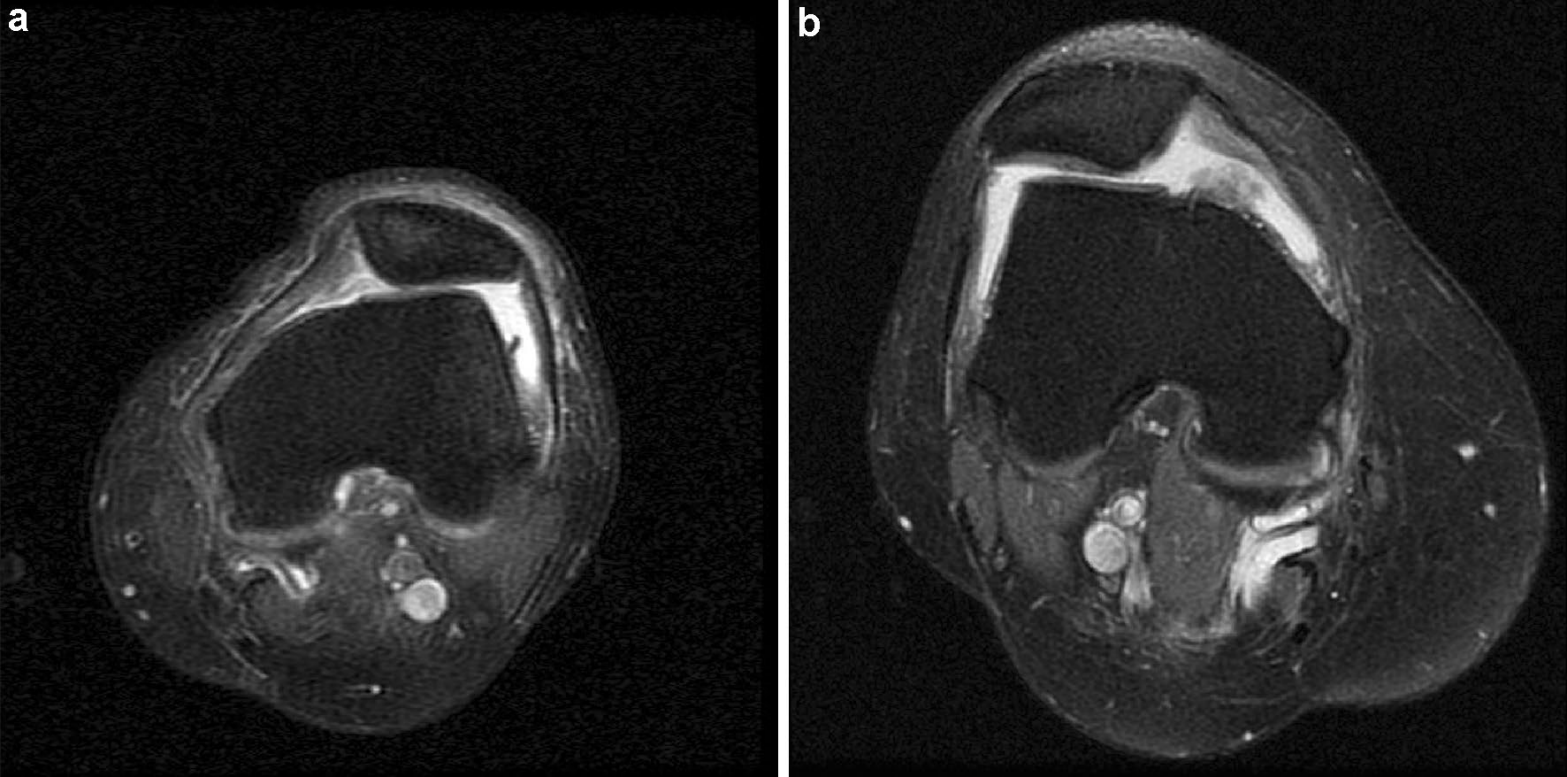
Axial PD-fatsat MRI (a section 3 cm above the knee joint). **a** Type B trochlear dysplasia is present. Note the flat trochlear groove and patellar subluxation. **b** Another patient was diagnosed with type B trochlear dysplasia, patellar subluxation, and patellar chondromalacia

Type B: flattened trochlear surface and a supratrochlear spur (Fig. 3a, b)

**Fig. 4:**
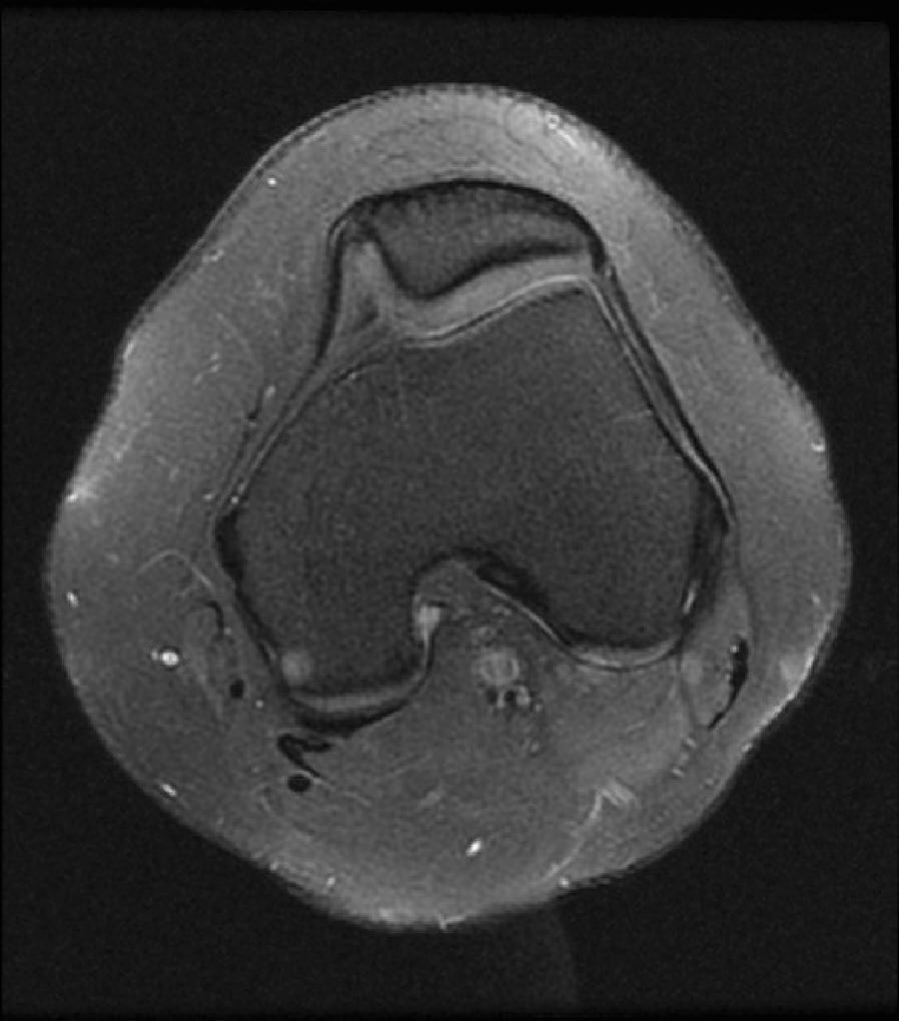
Axial PD-fatsat MRI (a Sect. 3 cm above the knee joint). Type C trochlear dysplasia is present. Note the trochlear fascial asymmetry, increased lateral convection, and medial facet hypoplasia

Type C: asymmetric trochlear surface; hypoplastic medial facet and convex lateral facet (Fig. 4)

**Fig. 5:**
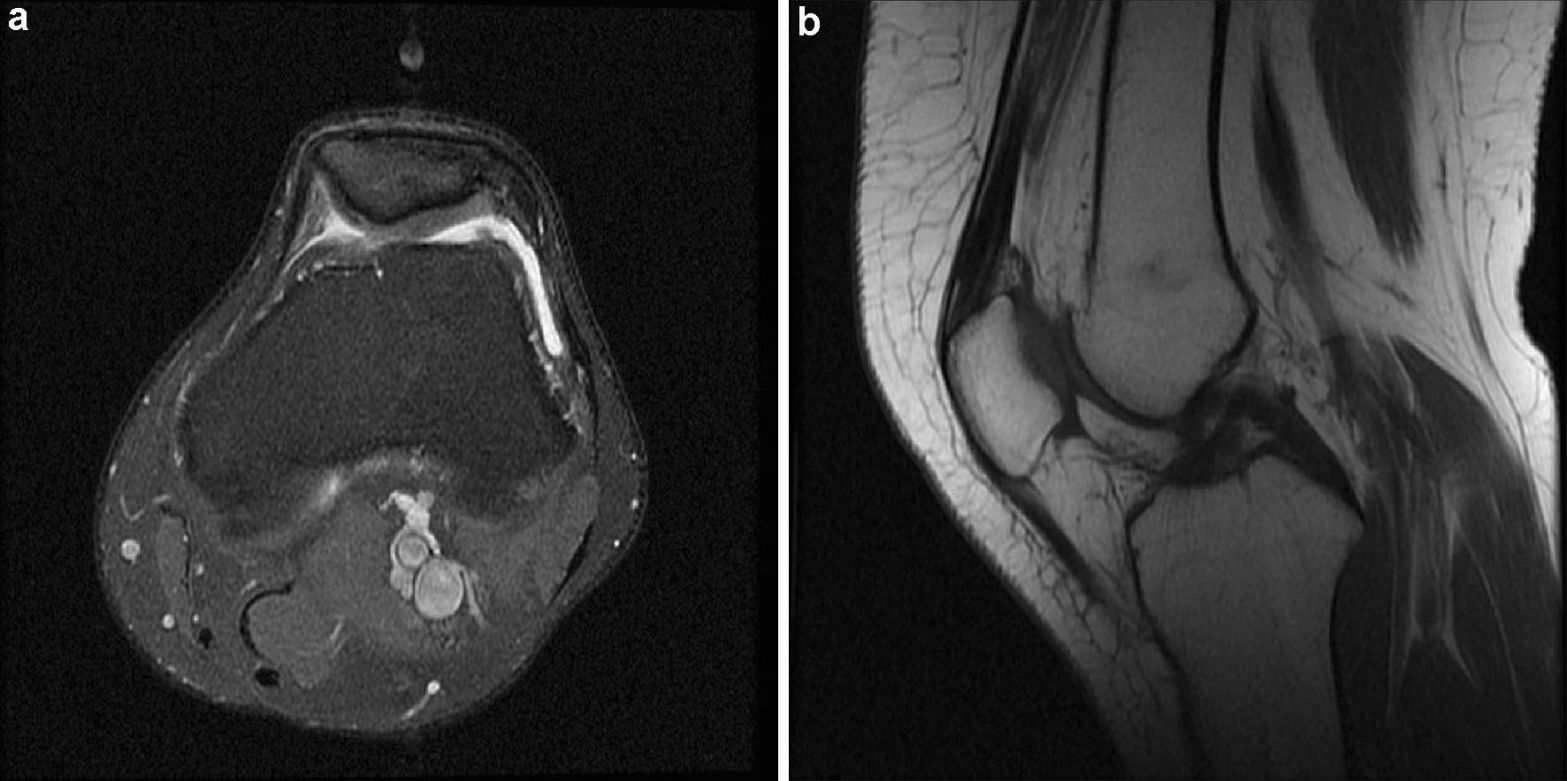
**a** Axial PD-fatsat MRI shows type D trochlear dysplasia. Note the trochlear surface asymmetry and hump. **b** Mid-sagittal T1-weighted fast spin echo MRI reveals a supratrochlear spur

Type D: humped shape; asymmetric trochlear surface with a supratrochlear spur (Fig. 5a)

The supratrochlear spur can be described as a ventral trochlear prominence (Pfirrmann et al. 2000). On a mid-sagittal image, the spur is seen as the distance between the anterior femoral cortical surface and the most prominent point of the trochlear surface (Fig. 5b). Measurements of >3 mm are accepted as indicative of a spur.

### Patellar height

The Insall and Salvati method (Insall and Salvati 1971) was used to measure the patella alta and patella baja. On MR imaging, the patellar and patellar tendon lengths of 0.8 and 1.3, respectively are considered normal on mid-sagittal images. The values for patella alta and patella baja were >1.3 and <0.8, respectively.

### Evaluation of the MPFLL/LPR ratio

**Fig. 6:**
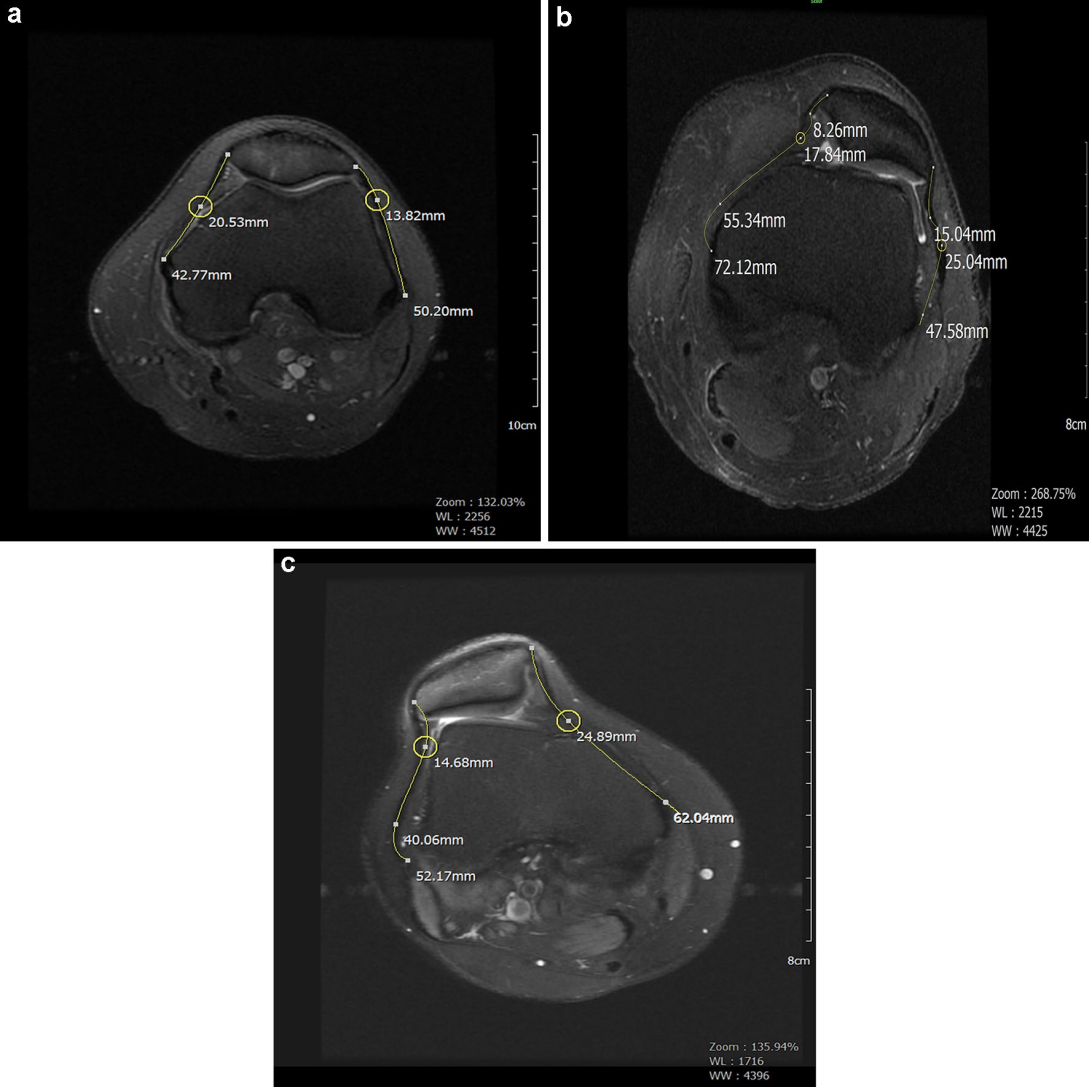
Medial patellofemoral ligament (MPFLL) and lateral patellar retinaculum length (LRR) measurements on axial PD-fatsat MRI crossing through the patellar center. **a** MPFL/LPR ratio of 42.77/50.20 = 0.85 was within normal limits. **b** This patient has type B dysplasia and patellar subluxation. MPFL/LPR ratio was 1.51, which was higher than the cutoff value. **c** This patient had type B dysplasia and patellar subluxation. MPRL/LPR ratio was 1.19

Axial sections passing through the center of the patella were used to determine the MPFLL/LPR ratio. The MPFLL ligament was measured between the patellar insertion and the femoral adductor tubercle. The LPR was measured between the patellar insertion of the retinaculum and the lateral epicondyle of the femur (Fig. 6a–c). Both retinacula exhibited a wide, fan-shaped extension from the patellar insertion region and distributed laterally among the muscle planes. The thickest parts of the ligament at the femoral and patellar insertion points were used for the measurements. This part of the study was conducted as an inter-observer study, and two blinded radiologists calculated the MPFLL/LPR ratio separately.

### Statistical analysis

A pilot study was first conducted as a power analysis. We predicted that we needed a minimum of 317 patients based on a 60 % frequency rate of trochlear dysplasia and 10 % margin of error, with an alpha error of 0.05 and a beta error of 0.05. The Shapiro–Wilk and single-sample Kolmogorov–Smirnov tests were used to test the normal distribution, and a histogram was drawn. Data are given as means and standard deviations; median, minimum, and maximum values; frequencies; and percentages based on their characteristics. Age and angle relations were tested using Spearman’s correlation test. Nominal variables were compared using the χ2 test with Yates correction and Fisher’s probability test. The odds ratio (OR) of trochlea types were obtained according to the “0” value.

For the MPFLL/LPR ratio, normality tests were conducted using the one-sample Kolmogorov–Smirnov test, histograms, box plots, and Q–Q (where Q = quantile) graphs. The correlation between the radiologists was evaluated using Pearson’s correlation test (for quantitative measurement values). Separate receiver operating characteristic (ROC) analyses were performed for the results of the radiologists. The comparison between the results according to the cutoff values found by the radiologists was assessed by the Z test. The concordance of these specialists with one another and with the gold standard was evaluated using the κ test. The specificity, sensitivity, positive predictive value (PPV), and negative predictive value (NPV) were also calculated for the two radiologists.

Non-parametric data were compared using the Mann–Whitney U test. The two-tailed significance level was adjusted to *P* < 0.05. All statistical analyses were conducted using NCSS10 software (www.ncss.com) and MedCalc 10.2 (medcalc.software.informer.com).

## Results

The study group included 317 patients [men/women 155 (48.9 %)/162 (51.1 %)] with a median age of 39.76 ± 11.89 years (17–73 years).

The patellar malalignment rate was 34.7 % (110/317 knees). There was no significant correlation between malalignment and sex (*P* = 0.131).

In all, 115 (36.2 %) patients had a normal trochlea, and 202 (63.7 %) had trochlear dysplasia. Altogether, 77 (38.1 %) had type A trochlear dysplasia, 82 (40.6 %) had type B, 38 (18.8 %) had type C, and 5 (2.5 %) had type D. Only type A trochlear dysplasia was more common among women (*P* = 0.002). There was no significant relation between age and the presence of trochlear dysplasia (*P* = 0.790).

In patients with a measurable sulcus angle, the mean trochlear angle was 142° in patients without patellofemoral malalignment and ≥146° in those with malalignment (*P* < 0.001). The frequency of trochlear dysplasia in conjunction with patellofemoral malalignment was 97.2 % (n = 107). Patellofemoral malalignment was found in 107 (52.9 %) patients with trochlear dysplasia and in 3 (2.6 %) patients with a normal trochlea (*P* < 0.001).

Malalignment frequency according to trochlear type was as follows: 31 (40.3 %) patients had type A dysplasia, 40 (48.8 %) had type B, 31 (81.5 %) had type C, and 5 (100 %) had type D. Of the five patients with type D dysplasia, two had patellar subluxation, and other three had patellar tilt. There was no significant difference between types A and B dysplasia in terms of patellar subluxation (*P* = 0.801). The patellofemoral malalignment rate, however, was significantly higher in patients with trochlear types C and D than in those with other trochlear types (*P* < 0.001).

Supratrochlear spurs were present in all five patients with type D trochlear dysplasia, whereas they were present in only 29 (35.3 %) of 82 patients with type B dysplasia.

The frequency of patella alta was increased in those with patellofemoral malalignment *(P* = 0.023). It was not significantly correlated with sex (*P* = 0.961). The frequency of patella baja was not increased in those with patellofemoral malalignment (*P* = 0.520), and it was not significantly correlated with sex (*P* = 0.121).

### MPFLL/LPR ratio

Cutoff evaluation results for the first radiologist conducted with ROC analysis revealed that the area under the curve (AUC) for an MPFLL/LPR ratio of 1.033 was 0.994, standard error 0.005; 95 % CI 0.969–0.999, sensitivity 99 %, specificity 94 %, PPV 95 %, NPV 99 %, and accuracy 97 %.

Cutoff evaluation results for the second radiologist conducted with ROC analysis revealed that the AUC for an MPFLL/LPR ratio of 1.041 was 0.984, standard error 0.009, 95 % CI 0.953–0.997, sensitivity 94 %, specificity 97 %, PPV 97 %; NPV 95 %, and accuracy 96 %. Comparison of the AUCs for the two radiologists showed that they were similar (*z* = 1.697, *P* = 0.090). A strong correlation was found between the calculations of the MPFLL/LPR ratio of the two radiologists (*r* = 0.90, P < 0.001).

The κ test was used to test the concordance between the MPFLL/LPR rate and patellofemoral malalignment. For the first radiologist, the values were κ = 0.93, SE = 0.027, *P* < 0.001. For the second radiologist, the corresponding values were κ = 0.91, SE = 0.031, and *P* < 0.001. The concordance of the researchers was investigated using the κ test and was found to be good (κ = 0.89, SE = 0.034, *P* < 0.001).

## Discussion

The two most common MRI findings in this study regarding patellofemoral malalignment were the presence of trochlear dysplasia and the high MPFLL/LPR ratio. The literature, in accordance with the results of our study, has suggested that trochlear dysplasia is the most important predisposing factor in patellar instability (Dejour et al. 1994). Dejour et al. (1994) found that the incidence of trochlear dysplasia was 85 % in patients with patellar instability. LaPrade et al. (2014) reported that 92.9 % (n = 118) of their patients with patellar instability had trochlear dysplasia. Compared with these results, the rate of trochlear dysplasia (97.2 %) associated with patellar malalignment in our study was higher than in the other studies. When the types of trochlear dysplasia were taken into account, Burmann (Burmann et al. 2011) reported incidences of 51.6 % type A, 25.4 % type B, 16.9 % type C, and 5.9 % type D-unlike in our study, where types B and C were more common than the other types.

The genetic origin of trochlear dysplasia was investigated in several studies (Glard et al. 2005; Balcarek et al. 2011). In that regard, we did not find a significant relation between trochlear dysplasia and age. We suggest that trochlear dysplasia is independent of age, which supports the effect of genetics on, and congenital development of, trochlear dysplasia, as suggested in the literature. Balcarek et al. (2010) reported that trochlear dysplasia is more frequent in women. In our study, only type A dysplasia was statistically significantly more frequent in women. In addition, there was no correlation between malalignment and sex in our study.

Patella alta is an important predisposing factor for patellar malalignment (Ward et al. 2007). When combined with other predisposing factors, patella alta leads to an increased risk of patellar dislocation (Diederichs et al. 2010). In accordance with the literature, our study showed that the frequency of patella alta was significantly increased in the subjects with patellofemoral malalignment. In contrast, patella baja had no important effect on patellar malalignment. Thus, among those with a patellar height pathology, only patella alta may be a predisposing factor.

During knee motion, especially between the initial 0°–20°, the balance between the medial and lateral retinacula keeps the patella in the trochlear groove (Desio et al. 1998). MPFL laxity, weakness, or damage and LPR shortness could impair patellar stability (Diederichs et al. 2010). Therefore, the initial surgery conducted for patellar instability is generally lateral retinaculum release, followed by MPFL reconstruction in the following years (LaPrade et al. 2014). Recurrence is seen in almost all patients who undergo lateral release alone, whereas a combination of those operations results in a better outcome in cases of patellar dislocation (Kolowich et al. 1990; Bedi and Marzo 2010). When considering the possible operative techniques used to repair patellar instability, surgeons take the medial and lateral retinaculum lengths into consideration. No measurement methods based on ligament length, however, have been reported in the literature to aid in the diagnosis of patellar malalignment. Hence, the MPFLL/LPR ratio in the quantitative evaluation of patellofemoral fitting problems is uniquely reported here. As the length of both ligaments may vary from subject to subject, we tried to make it independent of personal variability by using a ratio to find a cutoff point. We discovered that an MPFLL/LPR ratio cutoff value of 1.033–1.041 has very high sensitivity and specificity for diagnosing patellofemoral malalignment. Values > 1.041 indicate significant patellar malalignment. An MPFLL/LPR ratio of ≤ 1 indicates normal alignment. We suggest that the MPFLL/LPR ratio be used to diagnose patellar malalignment and that it could be used to guide the preoperative evaluation. Routine MRI, the current gold standard method, cannot define patellar malalignment adequately. Moreover, the more recent MRI measurements that could diagnose the malalignment have additional costs and require further software programs and experience, thereby increasing the time to diagnosis. The MPFLL/LPR ratio, which may be determined on routine MRI for diagnosing patellar malalignment, as we did in this study, is easily performed and allows measurements on just one axial MRI section.

The study had some limitations. We included patients with minor trauma or knee pain. A totally healthy population could not be examined for economic reasons. We used normal, static MRI, but weight-bearing MRI may be better for patellar localization. Even with these limitations, we believe that, using our novel technique with routine knee MRI to identify patellar malalignment, we could make a contribution to daily life.

In conclusion, patella alta and severe forms of trochlear dysplasia were detected frequently in association with patellar malalignment. The MPFLL/LPR ratio, with its high sensitivity, is a quantitative method based on ligament length to evaluate patellar malalignment. It was uniquely described here. The method is an easy, reliable measurement based on commonly used knee MRI without the need for additional software or experience.
